# Tau immunotherapy is associated with glial responses in FTLD-tau

**DOI:** 10.1007/s00401-021-02318-y

**Published:** 2021-05-05

**Authors:** Boram Kim, Bailey Mikytuck, Eunran Suh, Garrett S. Gibbons, Vivianna M. Van Deerlin, Sanjeev N. Vaishnavi, Meredith A. Spindler, Lauren Massimo, Murray Grossman, John Q. Trojanowski, David J. Irwin, Edward B. Lee

**Affiliations:** 1 Translational Neuropathology Research Laboratory, Department of Pathology and Laboratory Medicine, Perelman School of Medicine at the University of Pennsylvania, 613A Stellar Chance Laboratories, 422 Curie Blvd, Philadelphia, PA 19104 USA; 2 Center for Neurodegenerative Disease Research, Department of Pathology and Laboratory Medicine, Perelman School of Medicine at the University of Pennsylvania, Philadelphia, PA USA; 3 Penn Memory Center, Department of Neurology, Perelman School of Medicine at the University of Pennsylvania, Philadelphia, PA USA; 4 Parkinson’s Disease and Movement Disorders Center, Department of Neurology, Perelman School of Medicine at the University of Pennsylvania, Philadelphia, PA USA; 5 Penn Frontotemporal Degeneration Center, Department of Neurology, Perelman School of Medicine at the University of Pennsylvania, Philadelphia, PA USA

**Keywords:** Tau, Immunotherapy, Gosuranemab, Astrocytic tau, Astrocyte, Microglia

## Abstract

Progressive supranuclear palsy (PSP) and corticobasal degeneration (CBD) are neuropathologic subtypes of frontotemporal lobar degeneration with tau inclusions (FTLD-tau), primary tauopathies in which intracellular tau aggregation contributes to neurodegeneration. Gosuranemab (BIIB092) is a humanized monoclonal antibody that binds to N-terminal tau. While Gosuranemab passive immunotherapy trials for PSP failed to demonstrate clinical benefit, Gosuranemab reduced N-terminal tau in the cerebrospinal fluid of transgenic mouse models and PSP patients. However, the neuropathologic sequelae of Gosuranemab have not been described. In this present study, we examined the brain tissue of three individuals who received Gosuranemab. Post-mortem human brain tissues were studied using immunohistochemistry to identify astrocytic and microglial differences between immunized cases and a cohort of unimmunized PSP, CBD and aging controls. Gosuranemab immunotherapy was not associated with clearance of neuropathologic FTLD-tau inclusions. However, treatment-associated changes were observed including the presence of perivascular vesicular astrocytes (PVA) with tau accumulation within lysosomes. PVAs were morphologically and immunophenotypically distinct from the tufted astrocytes seen in PSP, granular fuzzy astrocytes (GFA) seen in aging, and astrocytic plaques seen in CBD. Additional glial responses included increased reactive gliosis consisting of bushy astrocytosis and accumulation of rod microglia. Together, these neuropathologic findings suggest that Gosuranemab may be associated with a glial response including accumulation of tau within astrocytic lysosomes.

**Supplementary Information:**

The online version contains supplementary material available at 10.1007/s00401-021-02318-y.

## Introduction

Progressive supranuclear palsy (PSP) is a sporadic neurodegenerative disorder that causes motor deficits including gait disturbance, postural instability, and vertical gaze palsy, as well as cognitive dysfunction [13]. PSP is rapidly progressive resulting in life-threatening complications such as dysphagia and pneumonia with a mean survival of 5 to 8 years [13]. There are no approved therapeutics that halt or attenuate disease progression.

PSP is a 4-repeat (4R) tauopathy and is a subtype of frontotemporal lobar degeneration with tau inclusions (FTLD-tau) which are primary tauopathies where tau protein accumulation is central to disease pathogenesis [15]. The neuropathological hallmarks of PSP include abnormal tau aggregates within neurons in subcortical nuclei and the brainstem, as well as within glia in the forms of tufted astrocytes and oligodendroglial coiled bodies [15]. Corticobasal degeneration (CBD) is also a 4R tauopathy characterized by more abundant tau accumulation in cerebral cortex regions and deep gray nuclei in the form of astrocytic plaques and threads [17]. CBD is clinically heterogeneous and can be the underlying neuropathology of some individuals with a clinical diagnosis of PSP.

Given that glial cells typically express low levels of endogenous tau [33], the accumulation of tau within glia may perhaps be due to the internalization of tau from extracellular milieu, consistent with the presence of tau in the interstitial fluid and cerebrospinal fluid of mouse and human brains [23, 32]. Furthermore, neuronal tau can be released into the extracellular space where tau aggregates appear to be transmissible between neighboring cells leading to the spread of pathologic tau throughout the brain [25]. Thus, extracellular tau has been proposed as a potentially disease-modifying target for tauopathies.

Gosuranemab (BIIB092) is a humanized IgG_4_ monoclonal antibody directed against the N-terminus of tau (residues 15–22), therefore able to recognize tau isoforms with an intact N-terminus including full length and N-terminal tau fragments [27]. Gosuranemab was derived from IPN002, a murine IgG_1_ monoclonal antibody and binds with high affinity to N-terminal tau secreted from Alzheimer’s disease (AD) patient-derived cortical neurons resulting in reduced neuronal hyperactivity [6]. Gosuranemab appears to exhibit high affinity to pathologic forms of tau from different tauopathies such as AD and PSP [27]. In addition, Gosuranemab attenuates tau seeding activity and decreases tau aggregates in vitro, and also significantly reduces N-terminal tau in both CSF and ISF in tau transgenic mice [27]. Collectively, these results suggested that Gosuranemab has the potential to bind extracellular tau and interrupt its transmission to neighboring cells, perhaps limiting the spread of tau.

With these promising preclinical studies, Gosuranemab was developed for clinical testing in humans. A phase 1 study showed a profound reduction in N-terminal CSF tau by up to 97% from healthy volunteers after a single dose [26]. A similar result was found in PSP patients in a phase 1b trial, showing decreased CSF N-terminal tau in the Gosuranemab-treated cohort compared with the placebo-treated cohort [5]. However, Gosuranemab did not achieve primary endpoints for a phase 2 trial for PSP (NCT#03068468).

The neuropathologic sequelae of Gosuranemab treatment have not been described. The goal of this study was to investigate whether therapy-related neuropathologic changes can be observed in individuals treated with Gosuranemab. We report here an autopsy study of three individuals who received Gosuranemab. While clearance of FTLD-tau aggregates was not observed in treated individuals, Gosuranemab treatment was associated with a glial response characterized by the accumulation of astrocytic tau within lysosomes, raising the possibility that anti-tau passive immunotherapy affects cerebral tau homeostasis.

## Materials and methods

### Autopsy cases

Autopsy materials were obtained from the University of Pennsylvania Center for Neurodegenerative Disease Research (CNDR) brain bank as described [29]. Demographic and diagnostic information on cases and controls are available in Table 1. Informed consent was obtained from all participants as approved by the University of Pennsylvania Institutional Review Board, and informed consent was obtained from next of kin for autopsy.

**Table 1:** Demographic and diagnostic information

Case #	Sex	Age of Onset	Age of Death	Clinical diagnosis	Primary Neuropathologic Diagnosis	Secondary Neuropathologic Diagnoses	ABC Scores			Study group
							A	B	C	
1	F	58	65	PSP	PSP with treatment effect	Low ADNC	1	1	0	Immunized PSP(Phase I, Study 251PP201, 2100 mg from MAD; 30 infusions up to 2 months prior to death)
2	F	61	67	PSP	PSP with treatment effect	Low ADNC	2	1	0	Immunized PSP(Phase I, Study 251PP201, 2100 mg from MAD; 22 infusions up to 1 month prior to death)
3	M	69	72	PSP	CBD with treatment effect		0	0	0	Immunized CBD(Phase II, Study 251PP301, 2000 mg; 10 infusions up to 1 month prior to death)
4	M	64	71	PSP	PSP	CVD, Low ADNC	1	0	0	Unimmunized PSP (Sibling of case #2)
5	M	70	73	bvFTD	PSP	Low ADNC	1	1	1	Unimmunized PSP
6	M	60	68	bvFTD	PSP	Low ADNC	1	0	0	Unimmunized PSP
7	F	69	79	PSP	PSP		0	1	0	Unimmunized PSP
8	M	n/a	64	PSP	PSP		0	0	0	Unimmunized PSP
9	M	65	70	DLB	PSP		0	1	0	Unimmunized PSP
10	M	74	77	PSP	PSP	Low ADNC	2	1	2	Unimmunized PSP
11	F	61	68	PSP	PSP		0	0	0	Unimmunized PSP
12	F	58	64	PSP	PSP		0	0	0	Unimmunized PSP
13	M	66	72	PSP	PSP		0	0	0	Unimmunized PSP
14	F	60	53	PSP	PSP		0	0	0	Unimmunized PSP
15	M	80	86	CBS	CBD		0	1	0	Unimmunized CBD
16	F	51	57	CBS	CBD		0	0	0	Unimmunized CBD
17	F	76	80	CBS	CBD	Intermediate ADNC	3	2	1	Unimmunized CBD
18	M	59	63	CBS	CBD		0	1	0	Unimmunized CBD
19	M	65	72	MSA	MSA		0	1	0	Aging control:ARTAG
20	M	66	72	bvFTD	FTLD-TDP	Low ADNC	1	1	0	Aging control:ARTAG
21	F	74	86	AD (probable)	Intermediate ADNC	LATE	2	2	0	Aging control:ARTAG
22	M	n/a	86	PD with dementia	LBD (limbic) and high ADNC	LATE	3	3	3	Aging control:ARTAG
23	M	66	82	PD	LBD (limbic)	Low ADNC	1	2	0	Aging control:ARTAG
24	M	61	77	PD with dementia	LBD (neocortical)	Low ADNC	1	2	0	Aging control:ARTAG
25	M	74	82	PD with dementia	LBD (neocortical)	Intermediate ADNC	2	2	3	Aging control:ARTAG
26	M	71	84	AD (probable)	AGD	Low ADNC	3	1	3	Aging control:ARTAG
27	M	74	85	PD with dementia	LBD (neocortical)	Intermediate ADNC	3	2	2	Aging control:ARTAG
28	M	n/a	84	Normal	Low ADNC		2	1	2	Aging control:ARTAG

*A score* Aβ/amyloid plaques by Thal phases, *B score* Neurofibrillary tangle score by Braak stage, *C score* Neuritic plaque score by Consortium to Establish a Registry for Alzheimer’s Disease (CERAD), *F* Female, *M* Male, *PSP* Progressive supranuclear palsy, *ADNC* Alzheimer's disease neuropathologic change, *MAD* Multiple ascending dose, *CBD* Corticobasal degeneration, *CVD* Cerebrovascular disease, *bvFTD* behavioral variant of Frontotemporal Dementia, *DLB* Dementia with Lewy bodies, *CBS* corticobasal syndrome, *MSA* Multiple system atrophy, *ARTAG* Aging-related tau astrogliopathy*, FTLD-TDP* Frontotemporal lobar degeneration with TDP-43 (transactive response DNA binding protein 43 kDa), *LATE* Limbic-predominant age-related TDP-43 encephalopathy, *PD* Parkinson’s Disease*, LBD* Lewy body disease, *AGD* Argyrophilic grain disease

### Immunohistochemistry and double immunofluorescence

Formalin-fixed, paraffin-embedded, 6 μm thick sections were examined from gray matter of cortical regions (middle frontal, anterior cingulate, superior and middle temporal, entorhinal, angular, and visual), hippocampus (dentate gyrus and cornu ammonis), amygdala, lentiform nuclei (putamen and globus pallidus), thalamus, substantia nigra, midbrain tegmentum, pons (locus coeruleus and pons basis), medulla, and cerebellum including the dentate nucleus. For immunohistochemistry, after deparaffinization and rehydration of the sections, endogenous peroxidase activity was blocked by incubation in methanol/hydrogen peroxide for 30 min at room temperature. Heat-induced or formic acid antigen retrieval procedure was then performed using citrate-based unmasking solution (Vector Laboratories, Burlingame, CA, USA) or 88% formic acid (Thermo Fisher Scientific, Waltham, MA), respectively. Sections were washed in 0.1 M Tris buffer, pH 7.6, blocked in 2% fetal bovine serum (FBS) in 0.1 M Tris buffer, and incubated with primary antibodies (Supplemental Table 1). After overnight incubation, sections were rinsed with 0.1 M Tris buffer, blocked in 2% fetal bovine serum (FBS) in 0.1 M Tris buffer. Thereafter, binding was detected with species-specific biotinylated secondary antibodies and developed with the Vectastain Avidin–Biotin Complex (ABC) kit (PK-6100, Vector Laboratories) and ImmPACT 3′-Diaminobenzidine (DAB, SK-4105, Vector Laboratories). Sections were counterstained with Harris’s hematoxylin (Shandon Harris Hematoxylin, ThermoFisher Scientific, Cheshire, WA, USA).

For double immunofluorescence, deparaffinized and rehydrated brain sections were washed in 0.1 M Tris buffer and blocked in 2% FBS in 0.1 M Tris buffer after heat-induced or formic acid antigen retrieval procedures. Sections were then incubated at 4 °C overnight with PHF1 in a combination with another primary antibody (Supplemental Table 1). On the second day, the sections were washed in 0.1 M Tris buffer, blocked in 2% FBS in 0.1 M Tris buffer, and then incubated with Alexa Fluor 488- and 568-conjugated secondary antibodies (1:1000, Invitrogen) for 1.5 h in the dark at room temperature. After washing in 0.1 M Tris buffer, the sections were counterstained with 0.3 µM 4′,6-diamidino-2-phenylindole (DAPI, D1306, Thermo Fisher Scientific) in phosphate-buffered saline, pH 7.4 (PBS, Life Technologies, Grand Island, NY), and rinsed with PBS three times for 5 min. Coverslips were then applied to the sections with Prolong Glass Antifade Mountant (P36980, Thermo Fisher Scientific).

### Quantification of Pathologic Change

PHF1 recognizes tau protein phosphorylated at serine residues 396 and 444. Semi-quantitative scores for tau inclusions were recorded for 18 PHF1-stained brain regions for each PSP case corresponding to 0 absent, 0.5 rare, 1 mild, 2 moderate, and 3 severe.

In addition, quantitative image analysis of area measurements was conducted on PHF1 and Iba1stained midbrain sections. Stained sections were scanned using a Leica Aperio AT2 scanner and the scanned image files were imported to QuPath software for digital image analysis [4]. The midbrain tegmentum was contoured as the region of interests (ROIs) with a downsample factor 3 and Gaussian sigma 1. Proper thresholding for positive staining was determined for each image and the percent of area occupied by DAB-labeled immunoreactivity was calculated using “positive pixel stain measurements.”

For cell counting, a bright field microscope (Leica DM LB2) connected with LAS version 4.13 was used for image acquisition. For quantification of tau-positive astrocytes, twelve fields from PHF1-stained middle frontal and angular cortex sections each case were acquired at 20 × objective magnification (0.290 mm^2^ per field) and exported to Adobe Photoshop CC 2020 (San Jose, CA). Similarly, for quantification of astrocyte numbers, twelve fields from GFAP and Sox9-stained midbrain sections in each case were obtained at 40× magnification (72,400 μm^2^ per field). In each case, the number of cells was manually counted without knowledge of which case received immunotherapy.

### Statistical analysis

Mann–Whitney U test, linear regression model, and Bland–Altman plot were performed using GraphPad Prism (GraphPad Software, Inc, San Diego, CA) with *p*-values < 0.05 considered statistically significant with the caveat that very few cases immunized cases are available for analysis. Additional statistical measures included median, range including maximum and minimum, and mean ± standard deviation (SD).

## Results

Post-mortem brain autopsies were conducted on three individuals were enrolled in phase I (case 1 and 2) or phase II (case 3) trials to test the safety and efficacy of Gosuranemab for individuals with possible or probable PSP (Table 1). All three individuals were randomized to treatment arms and continued to receive Gosuranemab during open label phase extensions ranging from 10 to 30 infusions up to 1 to 2 months prior to death. No serious adverse events or obvious clinical alterations were observed. Another post-mortem brain autopsy (case 4) was conducted on an individual clinically diagnosed with PSP who was the sibling of case 2 but who did not receive Gosuranemab. While PSP is typically sporadic, this kindred provided a unique opportunity to compare the neuropathology of two affected siblings who were differentially exposed to Gosuranemab. Neither sibling had mutations in microtubule-associated protein tau (*MAPT)* or valosin-containing protein (*VCP)* which are the only known genes linked to autosomal dominant forms of primary tauopathy [11].

**Fig. 1:**
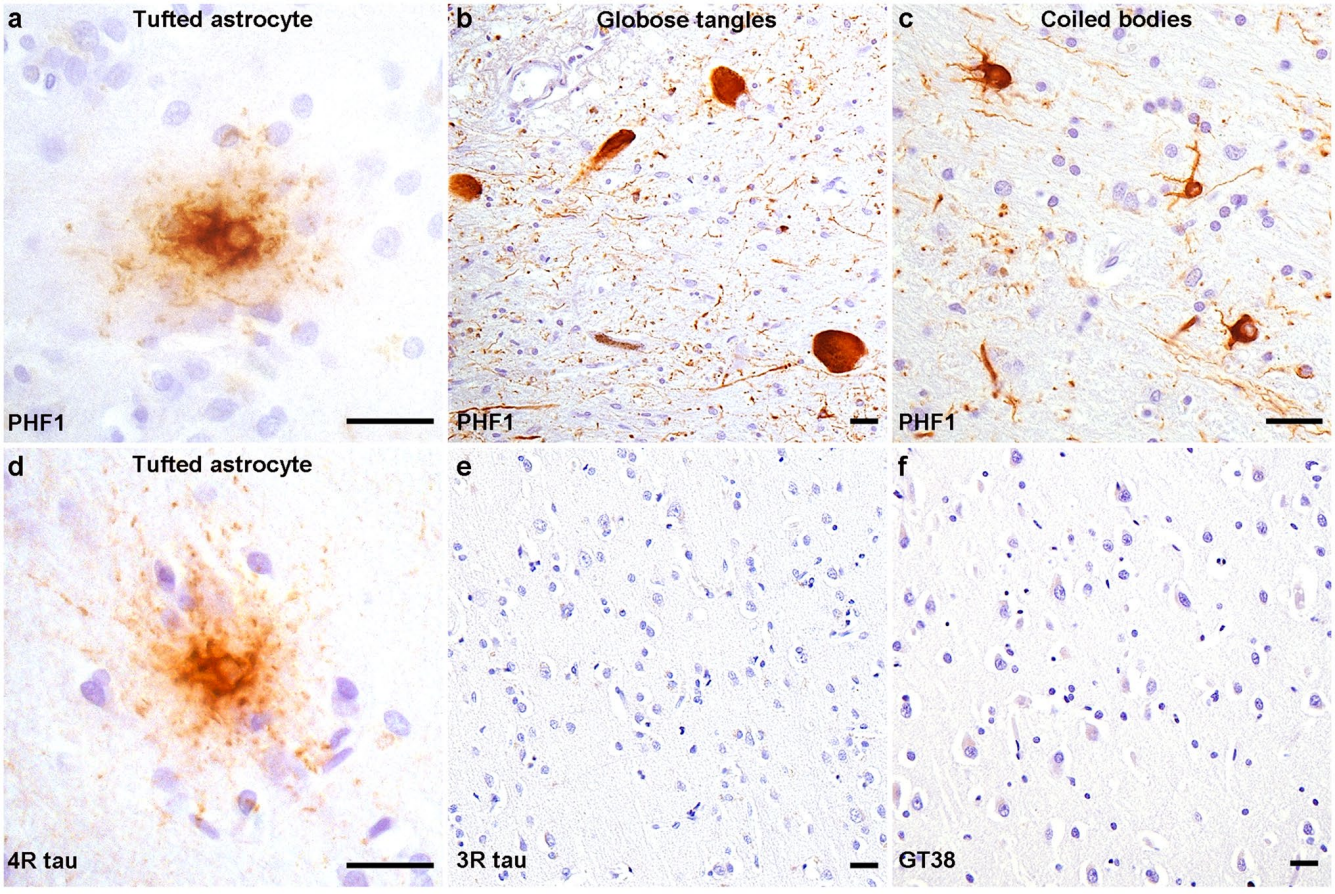
Tauopathy in unimmunized PSP. PHF1 immunohistochemistry shows **a** relatively few neocortical tufted astrocytes with densely packed tau-immunoreactive fibers in the proximal part of astrocytic processes. PHF1 staining of the brainstem shows **b** numerous globose tangles in the midbrain and **c** oligodendroglial coiled bodies in the white matter of the medulla. **d** 4R-tau specific antibodies recognize PSP-tau aggregates including tufted astrocytes in the middle frontal cortex. **e** 3R-tau specific and **f** GT38 antibodies do not detect PSP-tau aggregates across all sections. Scale bars = 25 μm

PHF1 immunohistochemistry performed on brain sections from the unimmunized sibling (case 4) detected phosphorylated tau-positive lesions with a cellular- and region-specific pattern indicative of stage 4 PSP [19]. Specifically, the cerebral cortex including the middle frontal cortex, angular gyrus, superior temporal gyrus, anterior cingulate, and visual cortex exhibited scant numbers of tufted astrocytes, characterized by tau-positive densely packed fibrils in the proximal processes (Fig. 1a). The medial temporal lobe contained minimal neuronal tau (Braak stage 0) with a moderate density of tufted astrocytes in the amygdala. Subcortical nuclei had a relatively high burden of tau inclusions including numerous tufted astrocytes. Similarly, the brainstem showed overall severe tau pathology with globose tangles (Fig. 1b), tufted astrocytes, and oligodendroglial coiled bodies (Fig. 1c). PSP-tau aggregates contained 4R-tau isoforms (Fig. 1d), but not 3R-tau isoforms (Fig. 1e). Immunohistochemistry using GT-38, a monoclonal antibody that recognizes a conformation present in 3R + 4R AD tau, was negative in PSP-tau inclusions (Fig. 1f). These findings were indicative of a neuropathologic diagnosis of PSP.

### Tauopathy associated with anti-tau passive immunization

**Fig. 2:**
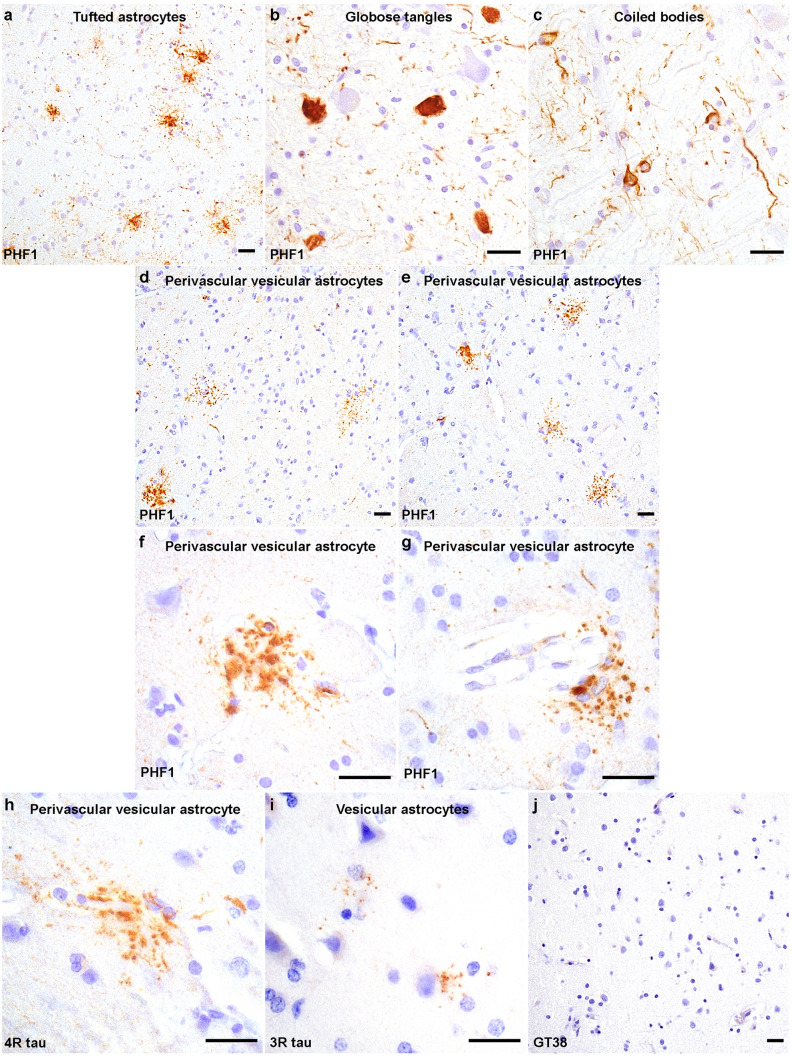
Tauopathy in immunized PSP cases. Tau pathologies detected by PHF1 shows typical PSP pathology including **a** numerous tufted astrocytes in the putamen, **b** moderate numbers of brainstem globose tangles and **c** white matter coiled bodies in the medulla. PHF immunohistochemistry of the neocortex from **d** immunized PSP case 1 and **e** case 2 shows perivascular vesicular astrocytes (PVAs) with atypical, dot/punctate tau immunoreactivity in astrocytic processes. **f**, **g** High magnification images show PVAs with vesicle-like tau immunoreactivity extending into the astrocytic processes around blood vessels **f** in the angular cortex of immunized PSP case1 and **g** in the middle frontal cortex of immunized PSP case 2. In the middle frontal cortex, PVAs are recognized using 4R-tau specific antibodies in both **h** immunized PSP case1 and (data not shown) case 2. **i** A few vesicular astrocytes are detected with 3R-tau specific antibodies in the middle frontal cortex of immunized PSP case 2. PVAs and PSP-tau inclusions are not detected upon immunostaining with the GT38 antibody in both (data not shown) immunized PSP case 1 and **j** case 2. Scale bars = 25 μm

PHF1 immunohistochemistry on immunized PSP cases (case 1 and 2) demonstrated tufted astrocytes, globose tangles, and oligodendroglial coiled bodies in a distribution and density that is typical of stage 5 (case 1) or 4 (case 2) PSP [19]. Passive immunotherapy in these two individuals did not result in clearance of PSP-tau inclusions, corroborated by digital image analysis of the percent area occupied by PHF1 immunoreactivity from midbrain sections comparing these two immunized PSP cases (1.03% ± 0.12) with 9 unimmunized PSP cases which included the unimmunized sibling (1.06% ± 0.17; Supplemental Fig. 1). Additional semi-quantitative grades for neuronal, astroglial and oligodendroglial tau inclusions across all brain sections did not reveal appreciable differences between immunized and unimmunized PSP cases (Supplemental Table 2). For example, there were few tufted astrocytes and neurofibrillary tangles throughout all cerebral cortex regions, whereas moderate numbers of tufted astrocytes and neuronal inclusions were present in the amygdala. Tufted astrocytes were numerous in the basal ganglia (Fig. 2a) where neuronal and oligodendroglial inclusions were also observed. Brainstem structures were heavily affected by tau pathology including globose tangles (Fig. 2b), coiled bodies (Fig. 2c) and neuropil threads. GT38 staining of the medial temporal lobe demonstrated a mild burden of AD neurofibrillary tangles pathology in both immunized PSP cases (Braak stage I).

Despite the absence of phospho-tau clearance (Supplemental Fig. 1 and Supplemental Table 2), both cases demonstrated what may represent treatment-related changes. Distinct, punctate tau immunoreactivity in astrocytic processes was found frequently throughout cerebral cortex gray matter, most prevalent in the angular gyrus (Fig. 2d) and middle frontal cortex (Fig. 2e). Higher magnification revealed vesicle-like tau immunoreactivity in the astrocytic processes mostly around blood vessels (Fig. 2f and 2g). Based on their morphology and microregional distribution, we termed this type of tau-positive astrocyte “perivascular vesicular astrocytes” (PVAs). PVAs contained 4R-tau isoforms (Fig. 2h), with very few vesicular astrocytes (VAs) being detected with anti-3R-tau antibodies seen only in the middle frontal neocortex of case 2 (Fig. 2i). The GT38 antibody did not detect either the PSP-tau inclusions or PVAs (Fig. 2j).

**Fig. 3:**
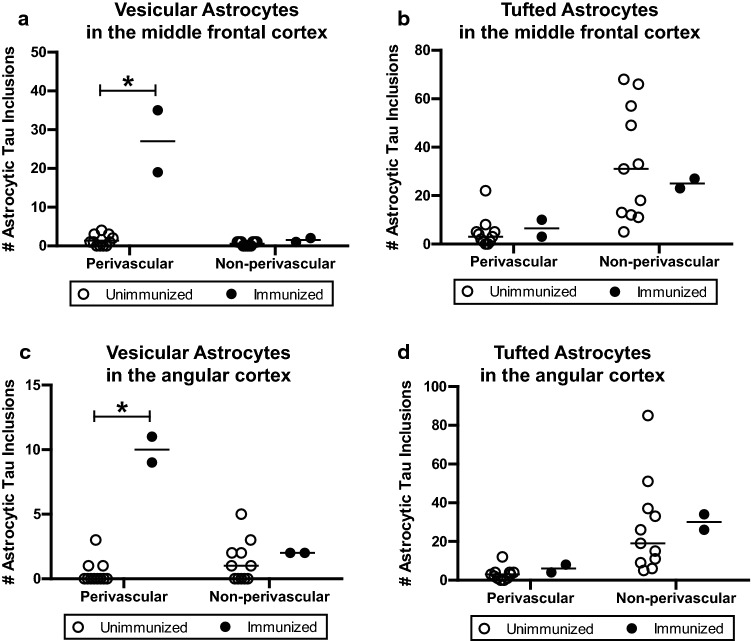
Quantitative analysis of astrocytic tau pathologies in immunized versus unimmunized PSP cases. Based on the morphology and perivascular localization, the number of PHF1-positive astrocytic tau inclusions were counted including perivascular vesicular astrocytes, non-perivascular vesicular astrocytes, perivascular tufted astrocytes, and non-perivascular astrocytes. Blinded counts corresponding to 12 images taken at 20× magnification are shown from **a**, **b** the middle frontal cortex and **c**, **d** the angular cortex. Median values within each group of cases are indicated. * p < 0.05, as determined by Mann–Whitney U test

On the basis of the morphologic and immunohistochemical profiles of astrocytic tau, we quantified neocortical astrocytic tau pathologies into the following categories: (1) perivascular vesicular astrocytes defined as vesicle-like tau immunoreactivity in the astrocytic processes around blood vessels; (2) non-perivascular vesicular astrocytes defined as vesicle-like tau immunoreactivity in the astrocytic processes not associated with a blood vessel; (3) perivascular tufted astrocytes defined as densely packed tau-immunoreactive fibers in the proximal part of astrocytic processes around blood vessels; (4) non-perivascular tufted astrocytes defined as densely packed tau-immunoreactive fibers in the proximal part of astrocytic processes not associated with a blood vessel. Blinded quantification of the middle frontal cortex demonstrated that the number of PVAs was significantly higher in the immunized group (*n* = 2, median = 27) compared to the unimmunized group (*n* = 11, median = 1, *p* = 0.013, Fig. 3a). There was no difference in non-perivascular VAs between the two groups (immunized group *n* = 2, median = 1.5; unimmunized group *n* = 11, median = 1;* p* = 0.218, Fig. 3a). Quantification of the same sections revealed no differences between immunized or unimmunized groups in the number of perivascular tufted astrocytes (immunized group *n* = 2, median = 6.5; unimmunized group *n* = 11, median = 3; *p* = 0.423, Fig. 3b) and non-perivascular tufted astrocytes (immunized group *n* = 2, median = 25; unimmunized group *n* = 11, median = 31; *p* = 0.923, Fig. 3b).

Analysis of the angular cortex yielded similar results. The quantity of angular cortex PVAs was higher in the immunized group (*n* = 2, median = 10) compared to the unimmunized group (*n* = 11, median = 0; *p* = 0.013, Fig. 3c). In contrast, there were no significant differences between immunized and unimmunized PSP cases in terms of the number of non-perivascular VAs (immunized group *n* = 2, median = 2; unimmunized group *n* = 11, median = 1; *p* = 0.346, Fig. 3c), perivascular tufted astrocytes (immunized group *n* = 2, median = 6; unimmunized group *n* = 11, median = 3; *p* = 0.205, Fig. 3d), and non-perivascular tufted astrocytes (immunized group *n* = 2, median = 30; unimmunized group *n* = 11, median = 19; *p* = 0.539, Fig. 3d). These results suggest that neocortical PVAs were associated with Gosuranemab immunization with the notable caveat that statistical interpretation is limited by the low number of available cases (Supplemental Fig. 2).

### Lysosomal tau accumulation in PVAs

**Fig. 4:**
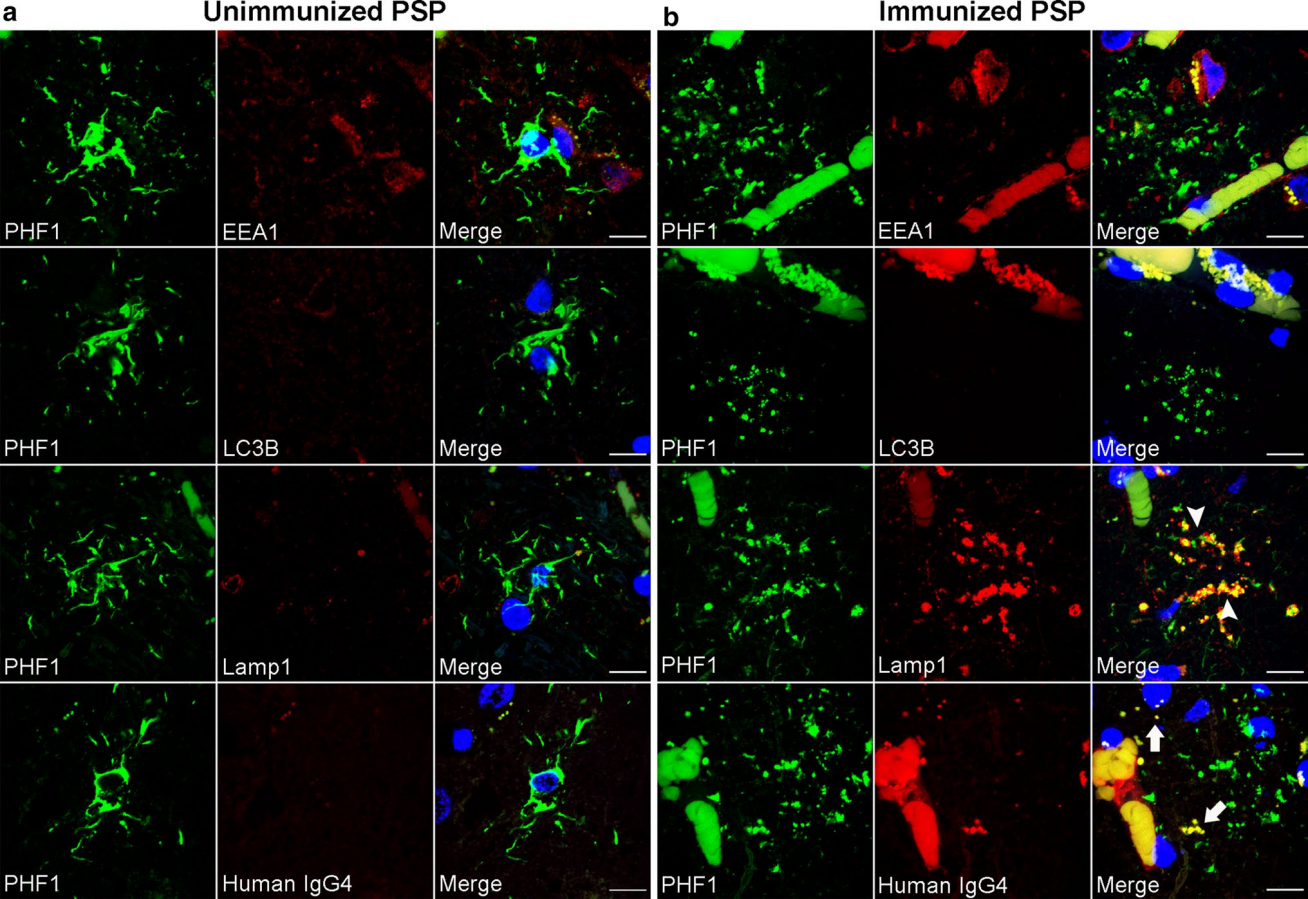
Lysosomal Tau Accumulation in Perivascular vesicular astrocytes. Double immunofluorescent staining was performed on middle frontal cortex sections from unimmunized (*n* = 6) and immunized PSP cases (*n* = 2). **a** Unimmunized PSP cases showed tufted astrocytes with no colocalization of PHF1-positive tau with endosomes (EEA1), autophagosomes (LC3B), lysosomes (Lamp1), or human IgG_4_ antibody (human IgG_4_). **b** Immunized PSP cases showed both tufted astrocytes and PVAs where the PVAs exhibited no colocalization of PHF1-positive tau with endosomes or autophagosomes. However, PVAs demonstrate nearly complete colocalization of tau-positive vesicles with lysosomes (Lamp1, arrowheads) with a subset of tau-positive vesicles showing colocalization with human IgG_4_ antibody (arrows). Scale bars = 10 μm

To identify the specific nature of the tau-positive vesicular structures in PVAs, double immunofluorescence staining was performed on middle frontal sections of immunized and unimmunized PSP cases with antibodies against PHF1 together with early endosome antigen 1(EEA1) as an endocytic marker, microtubule-associated proteins 1A/1B light chain 3B (LC3B) as an autophagosome marker, or lysosomal-associated membrane protein 1 (Lamp1) as a lysosome marker. As shown by confocal microscopy, tufted astrocytes in unimmunized PSP tau did not colocalize with EEA1, LC3B, or Lamp1-positive vesicles (Fig. 4a). Similarly, tau immunoreactivity in PVAs did not colocalize with EEA or LC3B in immunized PSP cases (Fig. 4b). In contrast, tau-positive vesicles in PVAs almost completely colocalized with Lamp1-positive vesicles (arrowheads, Fig. 4b). These findings suggest that immunotherapy-associated PVAs accumulate tau within lysosomes.

The perivascular localization of PVAs also raised the possibility that these astrocytes perhaps may be exposed to the peripherally administered Gosuranemab. To explore this possibility, double immunofluorescence microscopy showed that some PHF-1 positive vesicles in PVAs show immunoreactivity for human IgG_4_ (arrows, Fig. 4b) which was not seen in tufted astrocytes (Fig. 4a). While definitive localization of Gosuranemab within PVA lysosomes cannot be demonstrated here, these results raise the possibility that peripherally administered Gosuranemab may be taken up by perivascular astrocytes due to their proximity to the blood–brain barrier.

**Fig. 5:**
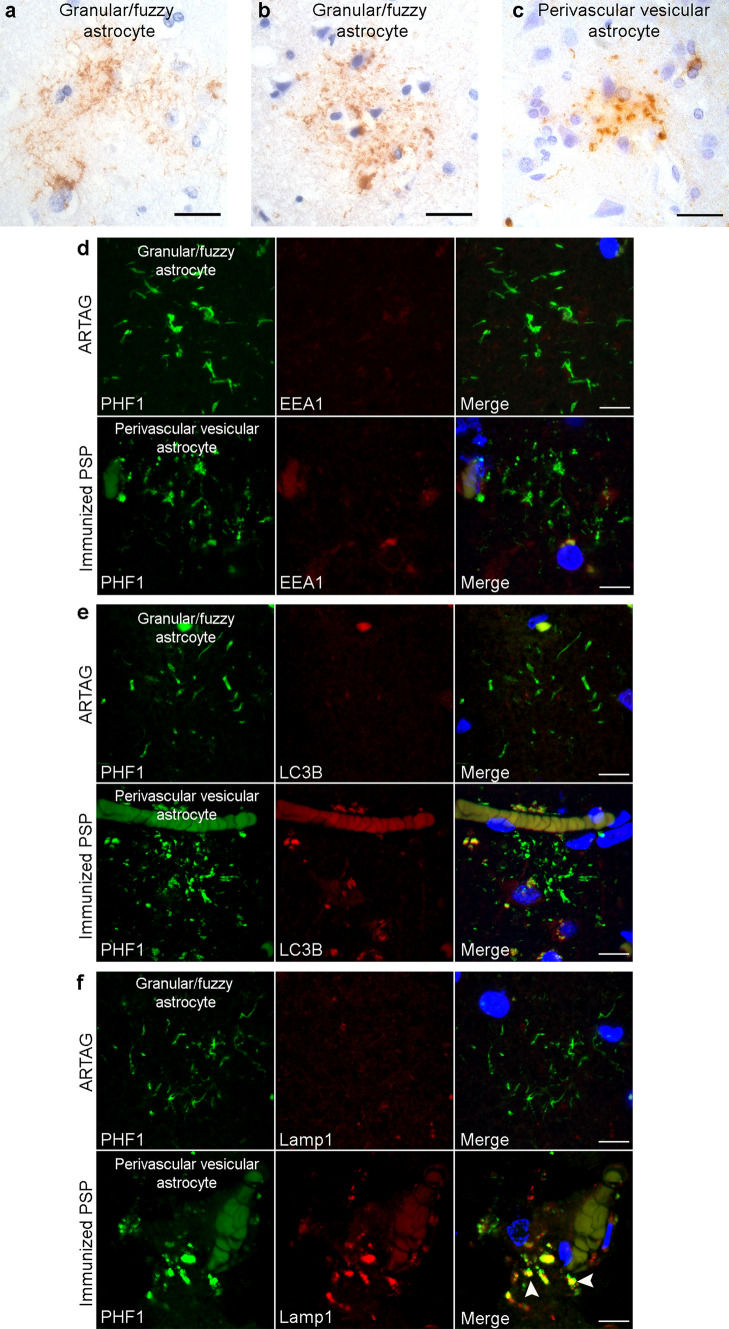
Morphologic and immunophenotypic differentiation of PVAs and GFAs. Immunohistochemistry was performed on neocortical sections from non-PSP cases with GFAs (*n* = 10) and immunized PSP cases (*n* = 2). **a** PHF1 immunohistochemistry of selected non-PSP cases showed GFAs with thin, short fiber-like tau immunoreactivity in branching processes of astrocytes seen in this section of middle frontal cortex and **b** short fibers combined with small granule-like tau immunoreactivity in astrocytic processes seen in this section of temporal cortex. **c** In contrast, PHF1-positive PVAs exhibit only granule-like structures without fibers as shown in this immunized PSP section of middle frontal cortex. Scale bars = 25 μm. Double immunostained neocortical sections of selected non-PSP cases show that PHF1 tau does not colocalize with **d** endosomes (EEA1), **e** autophagosomes (LC3B,) or **f** lysosomes (Lamp1). **d**–**f** PVAs from immunized PSP cases exhibited no colocalization of PHF1-positive tau with endosomes or autophagosomes but did show nearly complete colocalization of tau-positive vesicles with lysosomes (Lamp1, arrowheads). Scale bars = 10 μm

Granular/fuzzy astrocytes (GFAs) are a subtype of aging-related tau astrogliopathy (ARTAG), seen in the aging brain [18]. GFAs are characterized as astrocytic lesions with fuzzy fibrillar or fine granular tau immunoreactivity along astrocytic processes [18], preferentially found in gray matter relative to white matter [20]. These morphological and distributional features of GFAs are similar to those of PVAs in immunized PSP cases. Moreover, GFAs has been reported in primary tauopathies [20]. Therefore, we sought to differentiate PVAs from GFAs to demonstrate the specificity of PVAs to passive immunotherapy. We identified ten non-PSP cases with neocortical ARTAG for morphologic and immunohistochemical analysis of GFAs. In the PHF1-stained neocortical sections of the ARTAG control cases, two patterns of tau-positive GFAs were detected, the fuzzy fiber-like appearance in a combination with tiny granules (Fig. 5a) and, to a lesser extent, a more granule-like appearance (Fig. 5b), both of which were predominantly found in superficial neocortical layers. However, PVAs found in the immunized PSP were predominantly granular or vesicular without tau-positive fibers (Fig. 5c) across all neocortical laminae. Furthermore, PHF1 immunoreactivity in neocortical GFAs in normal control cases did not colocalize with EEA, LC3B, or Lamp1 (Fig. 5d–f), in contrast with neocortical PVAs in immunized PSP cases which extensively colocalized with Lamp1 (Fig. 5f). Thus, morphologic and immunophenotypic features are able to distinguish between PVAs and GFAs.

### Anti-tau immunotherapy and reactive gliosis

**Fig. 6:**
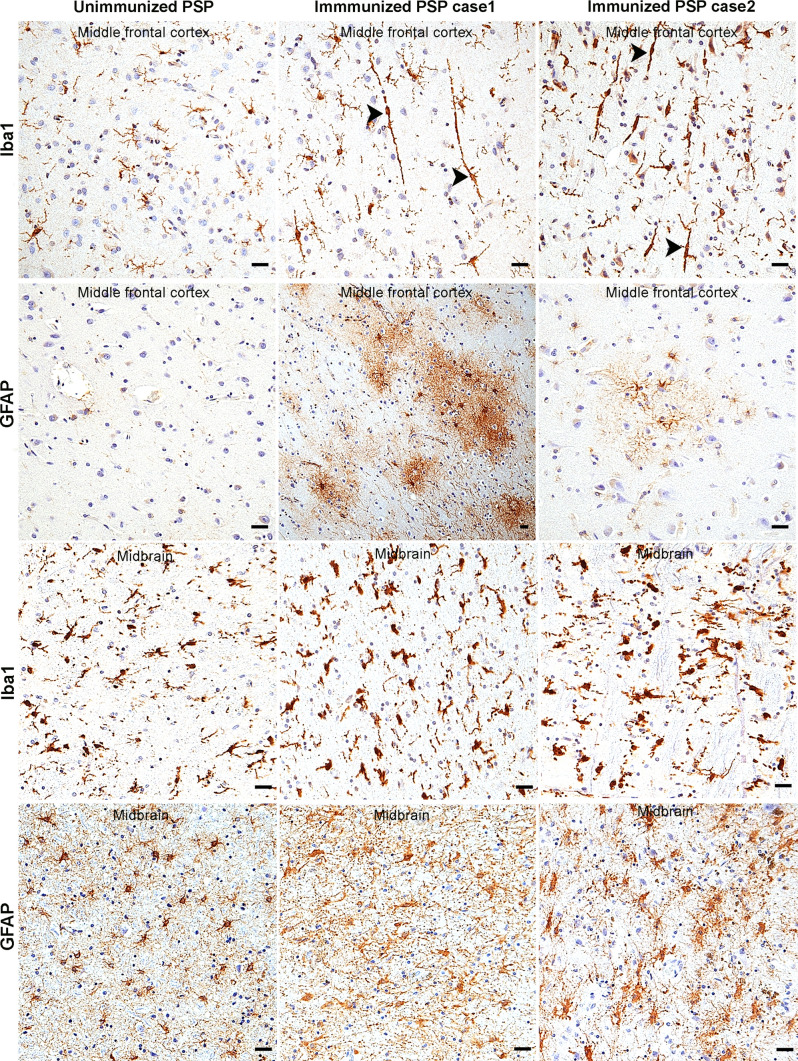
Immunotherapy is associated with an atypical reactive gliosis. Iba1 immunohistochemistry of middle frontal cortex (top row) of unimmunized PSP cases showed predominantly ramified microglia. Immunized PSP cases showed numerous rod-shaped microglia (arrowheads) with processes oriented perpendicular to the pial surface. GFAP immunohistochemistry (second row) of unimmunized PSP middle frontal cortex sections showed sparse reactive astrocytes, while immunized PSP cases showed mild (case 2) to severe astrocytosis (case 1) with atypical bushy morphology. Iba1 stain of brainstem (midbrain) showed reactive microgliosis which appeared to be more severe in immunized cases (third row). GFAP stain of brainstem (midbrain) showed astrogliosis with hypertrophied cellular processes in both the unimmunized and immunized PSP cases (bottom row). Scale bars = 25 μm

While the mechanisms by which Gosuranemab inhibits tau pathology in preclinical models is not entirely clear, the above results suggest that passive immunotherapy is associated with glial alterations. To further evaluate the glial responses associated with anti-tau immunotherapy in PSP, brain sections were immunostained for Iba1 to label microglia. Unimmunized PSP cases revealed ramified, homeostatic appearing microglia in neocortical regions with only a few scattered reactive microglia with cell body hypertrophy and thickened cell processes, consistent with the relative sparsity of tauopathy in these regions (Fig. 6, top row). In contrast, neocortical sections from both immunized PSP cases exhibited a proliferation of reactive bipolar or rod-shaped microglia with hypertrophied cell bodies and coarse processes that extended apically perpendicular to the pial surface and basally towards the subcortical white matter (Fig. 6, top row, arrowheads). Given the rarity of bipolar microglia in PSP [31], these observations suggest that anti-tau passive immunotherapy is associated with an atypical microglial response.

Neocortical sections were also stained with anti-glial fibrillary acidic protein (GFAP) antibody as an astrocytic marker. GFAP immunohistochemistry of unimmunized PSP cases showed no or rare reactive astrocytes across all neocortical regions (Fig. 6, second row). In contrast, there was mild (case 2) or severe (case 1) neocortical reactive astrogliosis (Fig. 6, second row) in immunized PSP cases. Notably, the reactive astrocytes observed in both immunized PSP cases exhibited an unusual bushy appearance which is different from typical reactive astrocytes which exhibit a more spidery to gemistocytic morphology.

Iba1 and GFAP immunostains were also performed on brainstem (midbrain) sections of both unimmunized and immunized PSP cases which revealed reactive microglia (Fig. 6, third row) and reactive astrocytes with hypertrophic cellular processes (Fig. 6, bottom row) in all PSP cases consistent with the high PSP-tau burden in these regions. Moreover, reactive glia appeared somewhat increased in the two immunized cases relative to unimmunized cases. Indeed, digital image analysis of the percent area occupied by Iba1 immunoreactivity showed an increase in microgliosis in immunized cases (Supplemental Fig. 3a). Evaluation of the percent area occupied by GFAP immunoreactivity in midbrain sections was difficult for several cases due to the high density of background glial processes. Therefore, we counted astrocyte numbers over multiple high-power fields based on either GFAP or Sox9 immunohistochemistry. Both immunostains revealed an increase in the number of midbrain astrocytes in immunized cases relative to unimmunized cases (Supplemental Fig. 3b–d) and were highly correlated with each other (Supplemental Fig. 3e; linear regression model, *n* = 13, *r*^2^ = 0.637, *p* = 0.001). However, counts based on GFAP immunohistochemistry were not statistically significant while counts based on Sox9 stain were statistically significant when comparing immunized and unimmunized cases, perhaps because the GFAP stain was less sensitive than the Sox9 stain (Supplemental Fig. 3f).

### Anti-tau immunotherapy in corticobasal degeneration

A post-mortem brain autopsy was performed on one additional individual with a clinical diagnosis of PSP who received Gosuranemab (Table 1, case 4). Rather than identifying PSP, this case exhibited abundant tau-positive neurites, astrocytic plaques, and coiled bodies in a distribution indicative of CBD. Again, there was no clear evidence that the immunotherapy was associated with clearance of CBD-tau lesions.

**Fig. 7:**
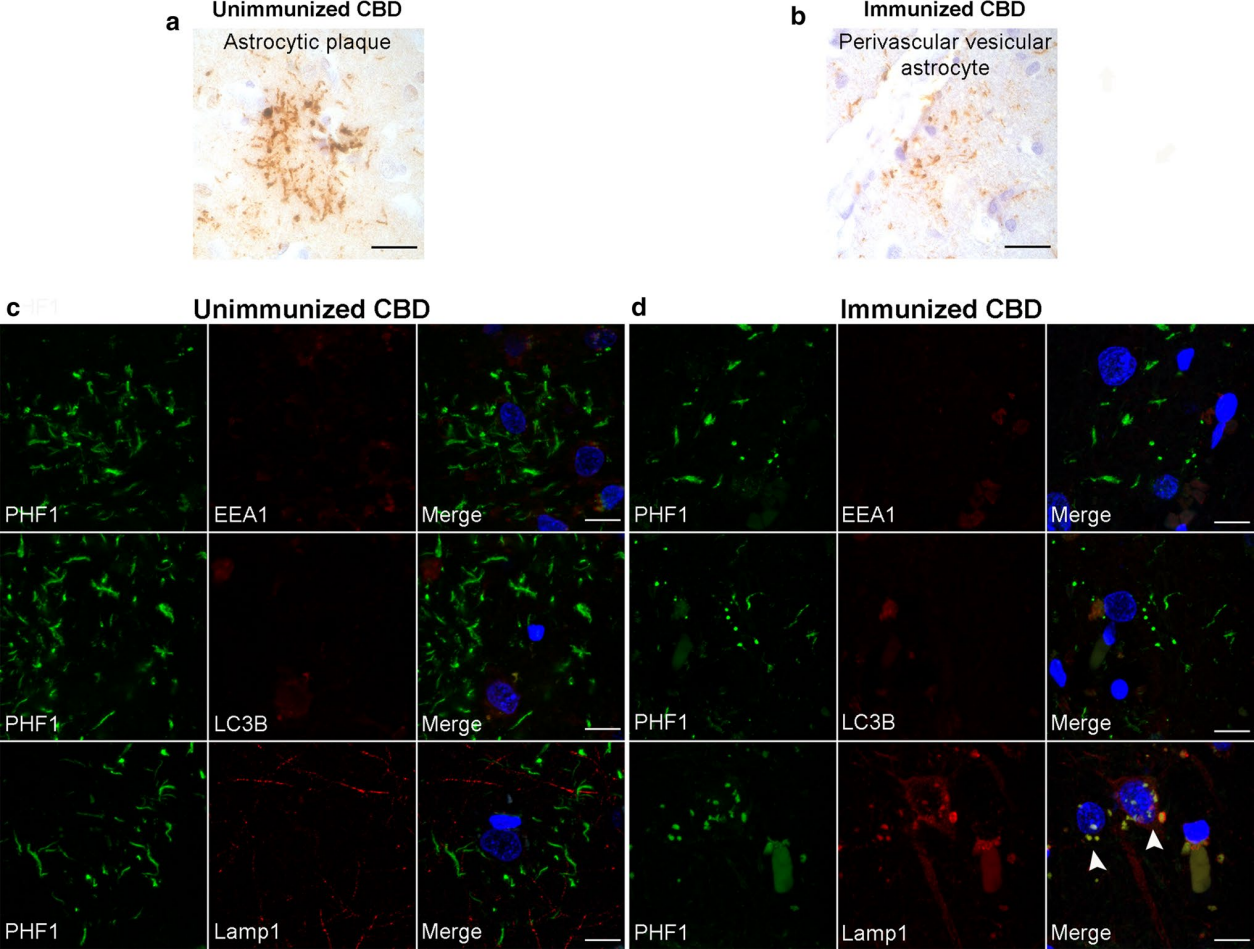
Immunotherapy treatment-related PVAs in corticobasal degeneration. Immunohistochemistry was performed on neocortical sections from unimmunized (*n* = 4) and immunized CBD cases (*n* = 1). **a** PHF1 immunohistochemistry of unimmunized CBD in middle frontal cortex shows astrocytic plaques with thick, short process-like tau-positive structures. **b** The immunized CBD case showed astrocytic plaques and threads in addition to PVAs with tau-positive, vesicular structures, as shown in this middle frontal cortex section. Scale bars = 25 μm. **c** Double immunostained neocortical sections of unimmunized CBD cases show that PHF1 tau in astrocytic plaques does not colocalize with endosomes (EEA1), autophagosomes (LC3B,) or lysosomes (Lamp1). **d** PVAs from the immunized CBD case exhibited no colocalization of PHF1-positive tau with endosomes or autophagosomes but did show extensive colocalization of tau-positive vesicles with lysosomes (Lamp1, arrowheads). Scale bars = 10 μm

Astrocytic plaques in unimmunized CBD cases consisted of tau-immunoreactive thick fibers extending into distal processes of astrocytes (Fig. 7a). Based on the above described finding of PVAs in immunized PSP cases, we determined whether similar PVAs could be seen in the case of CBD status-post immunotherapy. Although, the density CBD-tau in cerebral cortex regions consisting of threads and astrocytic plaques made identification of PVAs difficult, PVAs were ascertainable in cerebral cortex sections (Fig. 7b).

To corroborate this morphology-based assessment, double immunofluorescence microscopy of neocortex from a series of unimmunized CBD cases and the immunized CBD case was performed to differentiate astrocytic plaques and PVAs. Tau immunoreactivity in astrocytic plaques did not colocalize within EEA1 (endosomes), LC3B (autophagosomes), or Lamp1 (lysosomes, Fig. 7c). However, in the immunized CBD case, tau-positive PVAs were found which exhibited some colocalization between tau and Lamp1 lysosomal vesicles (Fig. 7d). Tau immunoreactivity in PVAs in this immunized CBD case did not colocalize with EEA1 or LC3B vesicles (Fig. 7d). These findings confirm that PVAs appear to be associated with anti-tau immunotherapy in the setting of FTLD-tau.

## Discussion

The overall purpose of this study was to assess the neuropathological effects of Gosuranemab in clinically diagnosed PSP patients. Gosuranemab treatment was not associated with obvious clearance of FTLD-tau aggregates. However, there was an unusual glial response consisting of bipolar/rod microgliosis, bushy astrocytosis, and the presence of tau accumulation within astrocytic vesicles in PVAs. This pattern of vesicular phospho-tau immunoreactivity was morphologically and immunophenotypically different from previously characterized astrocytic tau lesions including tufted astrocytes, GFAs and astrocytic plaques. Vesicular tau accumulation within PVAs was found in association with gray matter parenchymal blood vessels, most easily observed in PSP neocortical regions due to the relative paucity of FTLD-tau inclusions. Examination of an immunized case of CBD also revealed PVAs, suggesting that this astrocytic response is not limited to PSP. Tau-positive vesicular structures appear to be lysosomal with some vesicles demonstrated apparent immunoreactivity for human IgG_4_. We speculate that perivascular astrocytes may take up peripheral immunoglobulin due to their proximity to the blood–brain barrier, perhaps resulting in an antibody-dependent lysosomal accumulation of tau.

Tau immunotherapies are being tested as potential therapies to target extracellular tau to stop the progression of tauopathies. While passive immunization and active immunization reduced tau phosphorylation and oligomerization in tau transgenic animal models [7, 8, 16], it is unknown whether tau immunotherapy has an effect on tau pathology in human brain. Gosuranemab failed to demonstrate clinical efficacy in PSP, consistent with the absence of demonstrable clearance of PSP- or CBD-tau in immunized FTLD-tau cases. Importantly, our image-based quantification was based on PHF1 immunohistochemistry which recognizes tau phosphorylated at serine 395/404 and therefore mainly recognizes aggregated tau protein aggregates. Total tau levels, which could potentially be reduced as a result of Gosuranemab treatment, were not assessed in this study. Notably, these trials targeted possible or probable PSP which may represent an advanced stage of tauopathy. Conceptually, Gosuranemab targets extracellular tau and therefore may not be effective in clearing existing tau inclusions. Whether Gosuranemab is able to halt progression of disease by limiting the cell-to-cell transmission of tauopathy remains unclear.

Lysosomes are critical components of the autophagy and endosomal-lysosomal system that is essential to degrade aberrant proteins and maintain cellular homeostasis. Several tau antibodies promote tau degradation within lysosomes, resulting in decreased tau aggregates preclinical studies [7, 14, 21]. In this study, we found a nearly complete colocalization of PHF1-positive vesicles and Lamp1-positive vesicles in PVAs, suggesting that Gosuranemab may be associated with tau sequestration into lysosomes for degradation. As Gosuranemab recognizes the N-terminus of tau, a major form of extracellular tau [5, 27], we speculate that PVAs may represent an antibody-dependent internalization of extracellular tau whereby antibody-tau complexes are targeted to lysosomes for degradation. This would be consistent with previous studies showing cellular uptake of tau antibodies both in vivo and in vitro [1, 2, 9, 21]. Thus, further optimization of immunotherapy tau epitopes and facilitating the cellular uptake of tau represent opportunities for antibody-based therapeutic development.

Reactive astrogliosis is a common feature of tauopathies [22]. Its distribution correlates to neuronal tau rather than astrocytic tau [12, 28]. In contrast to experimental findings that tau antibodies reduced astrogliosis [10], we unexpectedly found upregulated astrogliosis in the neocortex and brainstem of immunized PSP cases. Notably, the bushy morphology of the reactive astrocytes in immunized PSP cases was unusual with extension of thin GFAP immunoreactivity into distal astrocytic processes. In contrast, reactive astrocytes associated with neurodegeneration typically exhibit a more gemistocytic to spidery morphology with GFAP accumulation in perikarya and proximal processes. In addition to increased microgliosis, morphologically atypical bipolar/rod microglia were also observed in immunized PSP cases. Such microglia have been noted in various neurological disorders including AD [3], traumatic and hypoxic brain injuries [24, 34], and encephalitis [30]. The function of bipolar/rod-shaped microglia are largely unknown. Bipolar/rod-shaped microglia are typically scarce in PSP [31]. The functional consequences of bushy astrocytosis and bipolar/rod microglisis in the setting of immunotherapy remain to be elucidated.

In conclusion, we describe a glial response in post-mortem human FTLD-tau brains associated with anti-tau passive immunization. An inevitable limitation of our post-mortem study is the assessment of tau and glial pathologies at a single end-stage time point. Although the unusual glial neuropathologic change observed here is striking, the low number of cases examined here is also a notable weakness of our study. Although Gosuranemab did not appear to elicit an obvious reduction of FTLD-tau aggregates, the presence of a glial response including the accumulation of phospho-tau within perivascular astrocytes suggests that anti-tau passive immunotherapy is associated with an alteration of tau homeostasis that perhaps requires further refinement to exert clinical benefit.

## Supplementary Information

Below is the link to the electronic supplementary material.

Supplementary file 1 (PDF 1551 KB)
