# Supplementary material for: Tau immunotherapy is associated with glial responses in FTLD-tau
Source: Acta Neuropathol. Author manuscript; Available in PMC 2021 Aug 1. (PMC8270872; doi:10.1007/s00401-021-02318-y)
Supplement: Supplementary Material [file NIHMS1706127-supplement-Supplementary_Material.pdf]

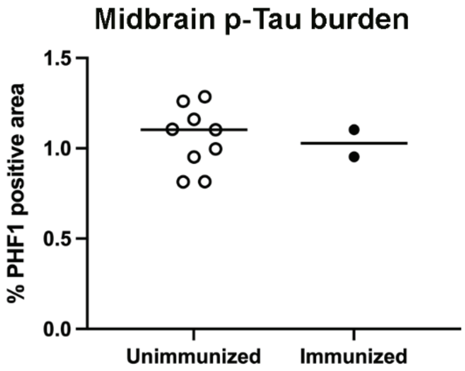

Mann-Whitney Test : P=0.746

	Unimmunized	Immunized
N	9	2
Median	1.10	1.03
Range	0.47	0.15
Maximum	1.29	1.10
Minimum	0.81	0.95
Mean ± SD	1.06 ± 0.17	1.03 ± 0.12

Supplemental Fig. 1 Quantitative analysis of total tau burden in immunized versus unimmunized PSP cases

The percent area occupied by PHF1 positive staining was calculated in the midbrain of unimmunized PSP (n=9) and immunized PSP (n=2). Unimmunized cases 5 and 7 were excluded from analysis due to high background which interfered with digital image analysis. Mann-Whitney U test revealed no differences in the median values between two groups. Descriptive results are shown in the summary table.

a # Vesicular Astrocytes in the middle frontal cortex

Perivascular

Mann-Whitney Test : P=0.013		
	Unimmunized	Immunized
N	11	2
Median	1	27
Range	4	16
Maximum	4	35
Minimum	0	19
Mean ± SD	1.36 ± 1.43	27.00 ± 11.31

Non-perivascular

Mann-Whitney Test : P=0.218		
	Unimmunized	Immunized
N	11	2
Median	1	1.5
Range	1	1
Maximum	1	2
Minimum	0	1
Mean ± SD	0.55 ± 0.52	1.50 ± 0.71

b # Tufted Astrocytes in the middle frontal cortex

Perivascular

Mann-Whitney Test : P=0.423		
	Unimmunized	Immunized
N	11	2
Median	3	6.5
Range	22	7
Maximum	22	10
Minimum	0	3
Mean ± SD	4.64 ± 6.27	6.50 ± 4.95

Non-perivascular

Mann-Whitney Test : P=0.923		
	Unimmunized	Immunized
N	11	2
Median	31	25
Range	68	4
Maximum	5	27
Minimum	63	23
Mean ± SD	33.00 ± 23.42	25.00 ± 2.83

c # Vesicular Astrocytes in the angular cortex

Perivascular

Mann-Whitney Test : P=0.013		
	Unimmunized	Immunized
N	11	2
Median	0	10
Range	3	2
Maximum	3	11
Minimum	0	9
Mean ± SD	0.46 ± 0.94	10 ± 1.41

Non-perivascular

Mann-Whitney Test : P=0.346		
	Unimmunized	Immunized
N	11	2
Median	1	2
Range	5	0
Maximum	5	2
Minimum	0	2
Mean ± SD	1.27 ± 1.62	2 ± 0

d # Tufted Astrocytes in the angular cortex

Perivascular

Mann-Whitney Test : P=0.205		
	Unimmunized	Immunized
N	11	2
Median	3	6
Range	12	4
Maximum	12	8
Minimum	0	4
Mean ± SD	3.09 ± 3.33	6.00 ± 2.83

Non-perivascular

Mann-Whitney Test : P=0.539		
	Unimmunized	Immunized
N	11	2
Median	19	30
Range	80	8
Maximum	85	34
Minimum	5	26
Mean ± SD	27.00 ± 24.06	30.00 ± 5.66

Supplemental Fig. 2 Descriptive statistics for the number of astrocytic tau inclusions in immunized versus unimmunized PSP cases. The sample size (N), median, range, maximum, minimum, mean ± standard deviation (SD), and p value as determined by Mann-Whitney test are presented for (a) perivascular and non-perivascular vesicular astrocytes in the middle frontal cortex, (b) perivascular and non-perivascular tufted astrocytes in the middle frontal cortex, (c) perivascular and non-perivascular vesicular astrocytes in the angular cortex, and (d) perivascular and non-perivascular tufted astrocytes in the angular cortex.

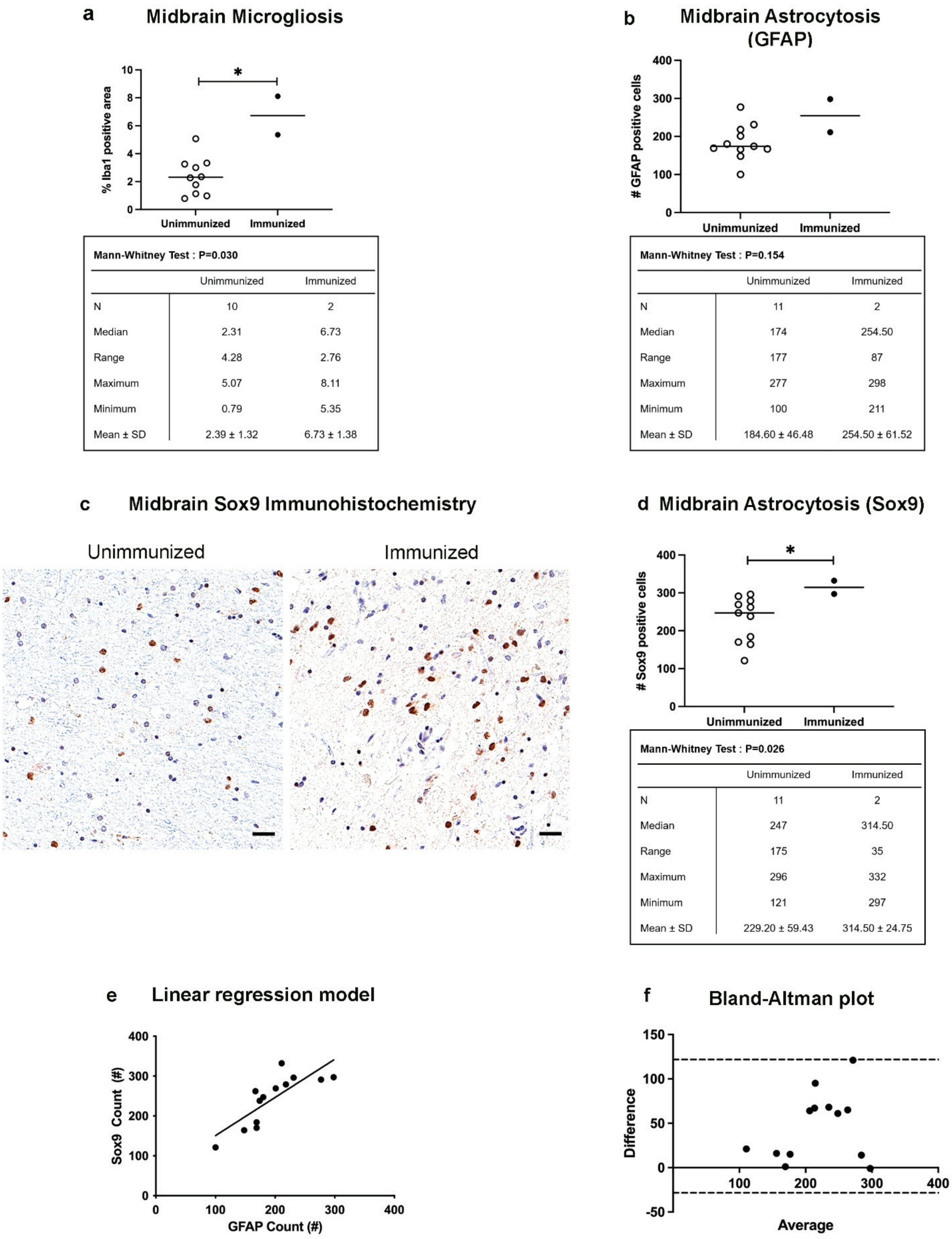

Mann-Whitney Test : P=0.030	Unimmunized	Immunized
N	10	2
Median	2.31	6.73
Range	4.28	2.76
Maximum	5.07	8.11
Minimum	0.79	5.35
Mean ± SD	2.39 ± 1.32	6.73 ± 1.38

Mann-Whitney Test : P=0.154	Unimmunized	Immunized
N	11	2
Median	174	254.50
Range	177	87
Maximum	277	298
Minimum	100	211
Mean ± SD	184.60 ± 46.48	254.50 ± 61.52

Mann-Whitney Test : P=0.026	Unimmunized	Immunized
N	11	2
Median	247	314.50
Range	175	35
Maximum	296	332
Minimum	121	297
Mean ± SD	229.20 ± 59.43	314.50 ± 24.75

Supplemental Fig. 3 Quantitative analysis of gliosis in immunized versus unimmunized PSP cases

The percent area occupied by positive thresholds for (a) Iba1 (n=10, immunized PSP, n=2, unimmunized PSP) was calculated. Case 5 exhibited high background which interfered with digital image analysis was excluded from the percent of Iba1 positive area measurements. (b) The number of GFAP positive cells in the midbrain of unimmunized PSP (n=11)

and immunized PSP (n=2) is shown. Representative images of (c) Sox9 immunohistochemistry and (d) quantification of Sox9 positive cells show increased number of astrocytes in the immunized PSP cases compared to unimmunized PSP cases. Median values within each group of cases are indicated and *$p<0.05$ was determined by Mann-Whitney U test. Descriptive results are presented in summary tables. (e) Linear regression model demonstrates a positive correlation between GFAP positive and Sox9 positive cell counts. (f) Bland-Altman plot shows 95% confidence limits of the bias for two counting measurements within two dotted lines, demonstrating that Sox9 was more sensitive in terms of identifying astrocytes.

Supplemental Table 1. Antigen and antibody list

Antigen	Antibody	Host	Concentration	Source
Phospho-tau (pS396 and pS404)	PHF1	mouse monoclonal	1:2000 for IHC<br>1:600 for IF	Gift from Peter Davies
4-repeat tau	Anti 4R-tau	mouse monoclonal	1:5000 for IHC	Cosmo Bio
3-repeat tau	RD3	mouse monoclonal	1:1000 for IHC	Millipore
AD-specific conformation tau	GT38	mouse monoclonal	1:1000 for IHC	Gift from CNDR
Iba1	Anti-Iba1	rabbit polyclonal	1:2000 for IHC	Wako
Sox9	Anti-Sox9	rabbit monoclonal	1:2000 for IHC	Abcam
GFAP	2.2B10	rat monoclonal	15000 for IHC	Gift from CNDR
EEA1	PA1-063A	rabbit polyclonal	1:100 for IF	ThermoFisher Scientific
LC3B	D11	rabbit polyclonal	1:1000 for IF	Cell Signaling Technology
LAMP1	Anti-LAMP1	rabbit polyclonal	1:450 for IF	Abcam
Human IgG_4	MA5-32169	rabbit polyclonal	1:1000 for IF	Invitrogen

IHC Immunohistochemistry, *IF* Immunofluorescence, *AD* Alzheimer's disease, *CNDR* Center for Neurodegenerative Disease Research, *GFAP* Glial fibrillary acidic protein, *EEA1* Early Endosome Antigen 1, *LC3B* Microtubule-associated proteins 1A/1B light chain 3B, *LAMP1* Lysosomal-associated membrane protein 1

Supplementary Table 2. Regional scoring of cellular tau pathologies in PSP

			Cortex-Grey matter					Medial Temporal Lobe				Subcortical Nuclei			Brain Stem					Cerebellum
Case	Group	Tau/Cell	FR	TE	AN	VI	CI	AM	HC DG	HC CA	HC EC	PU	GP	TH	SN	MB	LC	PB	MO	DN
1	PSP with	Neuron	0.5	0.5	1	0.5	1	2	0.5	0.5	0.5	0.5	1	2	3	2	2	2	2	2
	treatment	Astro	0.5	0.5	1	0.5	0.5	2	-	0	0	3	2	2	0	2	0	0.5	0.5	0
	effect	Astro-PVA	2	0.5	2	0.5	1	0.5	-	0	0.5	1	0.5	1	0	0.5	0	0.5	0.5	0
		Oligo	0.5	0	0.5	0.5	0.5	1	-	0	0	1	2	2	0	3	0	0.5	1	0.5
2	PSP with	Neuron	0.5	1	0.5	0	1	2	1	2	2	1	1	2	3	2	2	2	2	1
	treatment	Astro	0.5	0.5	0.5	0	1	2	-	0	0	3	2	2	0.5	1	0.5	0.5	0.5	0.5
	effect	Astro-PVA	2	0.5	2	0.5	1	0.5	-	0.5	1	1	1	0.5	0.5	0.5	0	0	0.5	0
		Oligo	0.5	0.5	1	0.5	0.5	0.5	-	0	0	0.5	1	2	0.5	2	0.5	1	0.5	0.5
4	Sibling of	Neuron	0.5	0.5	0.5	0	2	2	1	0.5	0.5	1	2	2	2	1	2	2	2	2
	case 2-PSP	Astro	0.5	0	0.5	0.5	0.5	1	-	0	0	3	3	1	1	2	0.5	1	0.5	0.5
		Oligo	0.5	0	0.5	0	1	0.5	-	0	0	0.5	2	2	0.5	2	0.5	1	0.5	1
5	PSP	Neuron	1	1	2	0	2	0.5	0.5	1	1	0.5	1	2	2	1	N/A	3	0	2
		Astro	2	1	3	0	1	0.5	-	1	1	2	1	1	0	1	N/A	0.5	0	1
		Oligo	2	0.5	1	0	1	0.5	-	0.5	0.5	0	1	2	1	1	N/A	0.5	0	1
6	PSP	Neuron	1	2	1	0.5	1	2	1	2	2	2	2	1	2	2	2	1	1	0
		Astro	2	2	2	0.5	1	2	-	2	2	2	1	1	0.5	1	0.5	0	0	0
		Oligo	0.5	1	1	0	0.5	2	-	3	2	2	1	1	1	1	1	1	1	0
7	PSP	Neuron	1	0.5	1	0.5	2	1	0.5	2	2	2	2	3	2	2	1	2	3	2
		Astro	0.5	0.5	1	0.5	0.5	0	-	0	0	3	2	1	0	0.5	0	0	0.5	0
		Oligo	0.5	0	0.5	0	0.5	0.5	-	0	0	1	2	3	0	2	0.5	1	2	1
8	PSP	Neuron	1	1	1	0.5	1	2	2	1	2	1	2	1	2	2	2	2	3	2
		Astro	3	1	1	0	3	3	-	2	2	2	3	3	0	1	0.5	0.5	2	0
		Oligo	1	3	2	0.5	1	1	-	0.5	1	1	2	3	0.5	3	0.5	1	3	1
9	PSP	Neuron	1	1	1	0.5	0.5	2	1	2	1	2	1	3	2	2	2	2	2	2
		Astro	1	0	0.5	0	0	1	-	0	0	1	2	2	0	0.5	0	0.5	0	1
		Oligo	0.5	0	0.5	0	0	0.5	-	0.5	0.5	1	2	3	0.5	3	0	1	2	2
10	PSP	Neuron	0	0.5	0.5	0	0	1	0.5	1	1	0.5	0.5	2	3	2	2	1	1	1
		Astro	2	1	2	1	0.5	2	-	0.5	1	2	2	3	2	2	0	0.5	0.5	0.5

		Oligo	0.5	0	0.5	1	0	0	-	0	0	0.5	2	2	2	2	0.5	0.5	0.5	0.5
11	PSP	Neuron	2	1	1	0	1	1	1	2	1	0	0.5	2	1	1	N/A	1	2	1
		Astro	3	2	3	1	3	1	-	1	0.5	2	1	3	2	2	N/A	0	0.5	1
		Oligo	1	1	1	1	1	1	-	0.5	0	1	2	3	1	2	N/A	1	0.5	1
12	PSP	Neuron	1	0.5	0.5	0.5	1	1	0.5	1	0.5	0.5	1	3	2	1	2	2	2	2
		Astro	1	0.5	0.5	0.5	1	0.5	-	1	0.5	3	3	3	2	3	2	0.5	1	1
		Oligo	1	0	0	1	1	0	-	0.5	0	1	2	3	2	1	1	1	3	2
13	PSP	Neuron	0.5	0.5	0	1	0.5	0.5	2	2	2	0.5	0.5	2	2	2	N/A	2	2	2
		Astro	2	1	0.5	2	2	0.5	-	2	2	3	2	2	2	2	N/A	1	1	2
		Oligo	0.5	0.5	0.5	0.5	0.5	0.5	-	1	1	1	1	1	1	1	N/A	1	2	2
14	PSP	Neuron	0.5	0.5	1	0	2	0.5	0	1	0.5	1	0.5	2	3	2	3	2	2	1
		Astro	0.5	0.5	2	0	2	0	-	0	0	2	1	2	2	3	1	1	1	1
		Oligo	1	0.5	3	0	1	0	-	0	0	0.5	2	2	1	3	0.5	0.5	1	3

FR frontal, *TE* temporal, *AN* angular, *VI* visual, *CI* anterior cingulate, *AM* amygdala, *HC* hippocampus, *DG* dentate gyrus, *CA* cornu ammonis, *EC* entorhinal cortex, *PU* putamen, *GP* globus pallidus, *TH* thalamus, *SN* substantia nigra, *MB* midbrain, *LC* locus coeruleus, *PB* pontine base, *MO* medulla oblongata, *DN* dentate nucleus, *Astro* astroglia, *Oligo* oligodendroglia. Semi-quantitative scoring system: 0 (none), 0.5 (rare), 1 (mild), 2 (moderate), 3 (severe).
